# Congenital diaphragmatic hernia: automatic lung and liver MRI segmentation with nnU-Net, reproducibility of pyradiomics features, and a machine learning application for the classification of liver herniation

**DOI:** 10.1007/s00431-024-05476-9

**Published:** 2024-02-28

**Authors:** Luana Conte, Ilaria Amodeo, Giorgio De Nunzio, Genny Raffaeli, Irene Borzani, Nicola Persico, Alice Griggio, Giuseppe Como, Donato Cascio, Mariarosa Colnaghi, Fabio Mosca, Giacomo Cavallaro

**Affiliations:** 1 Department of Mathematics and Physics “E. De Giorgi”, Laboratory of Biomedical Physics and Environment, Università del Salento, Lecce, Italy; 2 Advanced Data Analysis in Medicine (ADAM), Laboratory of Interdisciplinary Research Applied to Medicine (DReAM), Local Health Authority (ASL) Lecce and Università del Salento, Lecce, Italy; 3 Neonatal Intensive Care Unit, Fondazione IRCCS Ca’ Granda Ospedale Maggiore Policlinico, Milan, Italy; 4 Department of Clinical Sciences and Community Health, Università Degli Studi Di Milano, Milan, Italy; 5 Pediatric Radiology Unit, Fondazione IRCCS Ca’ Granda Ospedale Maggiore Policlinico, Milan, Italy; 6 Department of Obstetrics and Gynecology, Fondazione IRCCS Ca’ Granda, Ospedale Maggiore Policlinico, Milan, Italy; 7 ASST Fatebenefratelli Sacco, Ospedale Macedonio Melloni, Milan, Italy; 8 Department of Physics and Chemistry, Università Degli Studi Di Palermo, Palermo, Italy

**Keywords:** Congenital diaphragmatic hernia, Liver herniation, Segmentation, Artificial intelligence, Feature reproducibility, nnU-Net

## Abstract

Prenatal assessment of lung size and liver position is essential to stratify congenital diaphragmatic hernia (CDH) fetuses in risk categories, guiding counseling, and patient management. Manual segmentation on fetal MRI provides a quantitative estimation of total lung volume and liver herniation. However, it is time-consuming and operator-dependent. In this study, we utilized a publicly available deep learning (DL) segmentation system (nnU-Net) to automatically contour CDH-affected fetal lungs and liver on MRI sections. Concordance between automatic and manual segmentation was assessed by calculating the Jaccard coefficient. Pyradiomics standard features were then extracted from both manually and automatically segmented regions. The reproducibility of features between the two groups was evaluated through the Wilcoxon rank-sum test and intraclass correlation coefficients (ICCs). We finally tested the reliability of the automatic-segmentation approach by building a ML classifier system for the prediction of liver herniation based on support vector machines (SVM) and trained on shape features computed both in the manual and nnU-Net-segmented organs. We compared the area under the classifier receiver operating characteristic curve (AUC) in the two cases. Pyradiomics features calculated in the manual ROIs were partly reproducible by the same features calculated in nnU-Net segmented ROIs and, when used in the ML procedure, to predict liver herniation (both AUC around 0.85).

*          Conclusion*: Our results suggest that automatic MRI segmentation is feasible, with good reproducibility of pyradiomics features, and that a ML system for liver herniation prediction offers good reliability.

*          Trial registration*: https://clinicaltrials.gov/ct2/show/NCT04609163?term=NCT04609163&draw=2&rank=1; Clinical Trial Identification no. NCT04609163.

**Table:** 

**What is Known:** *• Magnetic resonance imaging (MRI) is crucial for prenatal congenital diaphragmatic hernia (CDH) assessment. It enables the quantification of the total lung volume and the extent of liver herniation, which are essential for stratifying the severity of CDH, guiding counseling, and patient management.* *• The manual segmentation of MRI scans is a time-consuming process that is heavily reliant upon the skill set of the operator.*	
**What is New:** *• MRI lung and liver automatic segmentation using the deep learning nnU-Net system is feasible, with good Jaccard coefficient values and satisfactory reproducibility of pyradiomics features compared to manual results.* *• A feasible ML system for predicting liver herniation could improve prenatal assessments and CDH patient management.*

**Supplementary Information:**

The online version contains supplementary material available at 10.1007/s00431-024-05476-9.

## Introduction

Congenital diaphragmatic hernia (CDH) is a rare congenital malformation characterized by a diaphragmatic defect that allows intrathoracic herniation of abdominal viscera, which affects normal lung development, leading to lung hypoplasia and postnatal pulmonary hypertension [1–3]. CDH affects 1 in 2500 births, but neonatal survival depends on several factors, such as defect side and size, herniated organs, associated anomalies, and gestational age at birth [4, 5]. Therefore, advanced imaging is crucial for a complete prenatal assessment and parental counseling. Combined evaluation of lung size, liver position, and defect side is conventionally accepted to stratify CDH fetuses in different groups, correlated with perinatal mortality and long-term morbidity [6, 7], and to guide prenatal intervention of fetal endoscopic tracheal occlusion (FETO) in selected cases [8, 9].

Fetal magnetic resonance imaging (MRI) enhances prenatal CDH evaluation through high anatomic specificity of the diaphragmatic defect, hernia location, content, and alteration in other fetal organs [10–12]. Therefore, it could be considered the most reliable technique to assess lung hypoplasia and calculate the observed/expected total fetal lung volume (O/E TFLV) [13]. It also permits a volumetric quantification of the intrathoracic hepatic parenchyma, expressed as liver herniation percentage (%LH) [14–16]. However, fetal MRI is an operator-dependent exam in which experience plays a key role, especially for segmentation, which is fundamental for accurate organ volume and shape assessment. However, general-usage medical image visualization software usually does not provide the physician with specific segmentation options, so the contouring work is still manual and prone to imprecision. Moreover, the broad spectrum of disease presentation poses additional challenges to the clinician [17].

Recently, the application of novel artificial intelligence (AI) technologies has been spreading in the neonatal field to support medical data analysis. Through the traditional machine learning (ML) approach and its modern deep learning (DL) extension, forecasting algorithms are built to predict specific outcomes, guide interventions, segment organs and vessels, and improve the overall quality of care [18–20].

However, these methodologies still need to be successfully applied to CDH newborns, so manual segmentation remains time-consuming and operator-dependent.

In CDH patients, building an automatic segmentation software could facilitate and standardize lung volume measurement, improve data collection accuracy, and create solid AI algorithms to predict postnatal outcomes.

In this study, we explored the possible application of a publicly available DL-based automatic segmentation system (nnU-Net) for automatic MRI contouring of the lungs and liver of fetuses with CDH. We then extracted pyradiomics standard features from the manual and the nnU-Net segmented ROIs to test the agreement between the two groups of features. Finally, a support vector machine (SVM) classifier was trained on shape features computed both in the manual and automatic segmentations of lungs and liver and employed to test the possibility of predicting liver herniation as a dichotomous variable (up/down).

## Materials and methods

This study represents an exploratory secondary analysis of the CLANNISH retrospective cohort study (Clinical Trial Identification no. NCT04609163) performed at Fondazione IRCCS Ca’ Granda Ospedale Maggiore Policlinico, Milan, Italy, involving the Fetal Surgery Center, Pediatric Radiology Service, Pediatric Surgery Unit, and Neonatal Intensive Care Unit (NICU) [21]. At the same time, the Department of Mathematics and Physics of the Università del Salento (Lecce, Italy) and the Department of Physics and Chemistry of the Università degli Studi di Palermo (Palermo, Italy) were involved in ML and DL data analyses and segmentation algorithms. A comprehensive description of the main study design has been previously published [21].

### Subjects

We enrolled 39 inborn patients, born between 01/01/2012 and 31/12/2020, with isolated CDH from singleton pregnancies, taken in charge at the Fetal Surgery Unit of the Fondazione IRCCS Ca’ Granda Ospedale Maggiore Policlinico (Milan, Italy) before the 30th week of gestation. The only exclusion criterion was a pre- or postnatal diagnosis of non-isolated CDH.

### Data collection

A retrospective data collection of clinical and radiological variables from newborns’ and mothers’ medical records was performed for eligible patients (Astraia, Astraia Software GmbH, Ismaning, Germany; NeoCare, GPI SpA, Trento, Italy). In addition, the native sequences from fetal MRI were collected, with separate acquisition for the lungs and liver.

### Manual segmentation of lung and liver volumes

The imaging software used was Synapse PACS and Synapse 3D (FUJIFILM Medical Systems Lexington, MA, US). Lung volumes were calculated on the T2 HASTE sequences, selecting the best image quality plane without motion-induced artifacts [22]. Liver volumes were calculated on T1 VIBE sequences [23]. A pediatric radiologist with 15 years of experience in fetal MRI performed the manual segmentation of lung and liver volumes. In each slice, left and right lung and liver areas were determined separately by tracing freehand regions of interest (ROIs), excluding the pulmonary hila and mediastinal structures. The areas were automatically added to obtain the entire organ volume, multiplied by the sum of slice thickness and intergap by the software.

The DICOM files were then anonymized, converted to the NIFTI format for easier manipulation, and fed to the segmentation pipeline.

### Segmentation with no-new-Net (nn-NET)

A publicly available segmentation pipeline based on DL achieved automatic lung and liver MRI segmentation. The pipeline was the no-new-Net (nn-NET), a specialized DL framework for medical image segmentation [24]. The framework is based on the U-Net architecture, a popular convolutional neural network that is particularly effective for biomedical image segmentation. It was developed to address the challenge of designing neural network architectures well-suited for various medical imaging tasks without requiring manual configuration or architectural modifications for each new task. nnU-Net automatically adapts its architecture to the specific characteristics of the dataset. It analyzes the dataset and decides on the most appropriate network architecture, preprocessing steps, and training strategies. This includes decisions about the network depth, convolutional kernel sizes, and the number of feature maps. This automation reduces the need for manual tuning and expert knowledge, making high-quality segmentation accessible even to those who might not be specialists in deep learning or medical image analysis. This network can achieve good segmentation results even with datasets of limited size. The nnU-Net segmentation pipeline is organized in several steps: (1) dataset structuring to a format compatible with the software; (2) the extraction of a dataset “fingerprint” containing dataset-specific properties, used to build various 2D/3D configurations, among which the best is “3D cascade”; (3) model training and validation, which we performed in the default fivefold cross-validation scheme. The software automatically gives Sørensen–Dice and Jaccard coefficients for segmentation quality evaluation. We ran the pipeline on a Server Supermicro 2023US-TR4, 2 CPU AMD Rome 7282 16C/32 T 2.8G 64 MB, equipped with 256 GB RDIMM DDR4 RAM and GPU Nvidia Tesla V100 32 GB HBM2 PCIe 3.0 (property of INFN, the Italian National Institute for Nuclear Physics, branch of Lecce). A cross-validation fold of each configuration took about 1 full day of calculations.

### Radiomics features

After segmentation, several standard 3D radiomics features were calculated. Pyradiomics was chosen for feature calculation [25]. This software package is freely available and allows the computation of many variables both from the original images and after preprocessing by various filters (e.g., wavelets or LoG, Laplacian of Gaussian). It also allows automatic reslicing with a chosen interpolator. The computed features, a subset of those available in pyradiomics, and after removing some correlated ones, are listed in Table 1. For gray level co-occurrence matrix (GLCM) and neighborhood gray tone difference matrix (NGTDM) calculation, only pixel pairs separated by a distance of 1 pixel were considered.

**Table 1:** Pyradiomics features (11 for shape, 17 for 1st order, and five groups for a total of 75 variables for higher-order features; the overall number of features is 103). Only features from the original images (no preprocessing) were considered

Shape	1st order	2nd order and up
Mesh volume, voxel volume, surface area, surface volume ratio, sphericity, maximum 3D diameter, major axis length, minor axis length, least axis length, elongation, flatness	Energy, total energy, entropy, 10th percentile, 90th percentile, interquartile range, minimum, maximum, mean, median, mean absolute deviation, robust mean absolute deviation, root mean squared, variance, skewness, kurtosis, uniformity	glcm, glrlm, glszm, gldm, ngtdm

*glcm* gray-level co-occurrence matrix, *glrlm* gray-level run length matrix, *glszm* gray-level size-zone matrix, *gldm* gray-level dependence matrix, *ngtdm* neighborhood gray tone difference matrix

The MR images were preliminarily resized to all have the same voxel size of 1 × 1 × 1 mm^3^; the sitkBSpline interpolator was used for this purpose.

### Reproducibility of pyradiomics features

In order to test if the features calculated from manually and automatically segmented ROIs had similar values, a Wilcoxon rank-sum test and tests based on the intraclass correlation coefficients (ICCs) were performed. ICC is a statistical measure ranging from 0 to 1, with values close to 1 representing stronger feature reproducibility in segmentations. McGraw and Wong [26] defined 10 forms of ICC. In this study, we calculated the interrater reliability by employing a two-way mixed effect, absolute agreement, single rater/measurement model considering the variation between two or more raters who evaluate the same group of subjects (Eq. 1) [27]:

1 $${\text{ICC}}= \frac{{MS}_{R}-{MS}_{E}}{{MS}_{R}+\left(k-1\right){MS}_{E}+ \frac{k}{n}({MS}_{C}- {MS}_{E})}$$

where *MS*_*R*_ is the mean square for rows, *MS*_*E*_ is the mean square error, *MS*_*C*_ is the mean square for columns, *k* is the number of observers involved, and *n* is the number of subjects.

A freely available code was used for ICC computation [28]. According to ICC values, we stratified the features into four groups as having excellent (ICC >  = 0.75), good (0.60 <  = ICC < 0.75), fair (0.40 <  = ICC < 0.60), or poor (ICC < 0.40) reproducibility [29]. The reproducibility within groups of features was also assessed using the Wilcoxon rank-sum test with a *p* value threshold set at 0.05.

### Prediction of liver herniation by machine learning

To test the possibility of predicting liver herniation as a dichotomous variable (up/down), several ML forecasting algorithms were implemented in the Matlab environment and Python, according to the experimenters’ convenience, using features calculated by pyradiomics. Several ML approaches were tested, such as decision trees, linear and non-linear artificial neural networks (ANN), and support vector machines (SVM) with various standard kernels.

Decision trees are a widely used method in ML, for both classification and regression tasks. A decision tree works by breaking down the classification procedure into a series of steps, each represented by a tree node (or leaf), so that an associated decision tree is incrementally developed. Each step asks a question that has a “yes” or “no” answer and redirects the flow towards different branches as you move down to another node or a tree lead, depending on the answer. The path from root to the final leaves (the classes) gives the overall classification rule. ANNs are inspired by the structure of the human brain. They consist of layers of interconnected nodes, known as neurons, which process information. Each connection between neurons has a weight that adjusts as the ANN learns from data. This structure allows ANNs to learn complex patterns and make predictions or decisions. ANNs can be linear or non-linear, depending on how these nodes and layers are arranged and interact. In simple terms, ANNs are like complex webs that learn to recognize patterns from the data they are trained on. Support vector machines (SVM) are another method used for classification and regression tasks. SVMs work by finding the best boundary that separates data into classes. This boundary is chosen to maximize the margin, or distance, between the boundary and the closest data points from each class, known as support vectors. SVMs are efficient in high-dimensional spaces and are versatile, as they can use various kernels (mathematical functions) to transform data so that a non-linear boundary can be used linearly.

For this part, only left-sided CDH patients were considered because of their larger numerosity, homogeneity, and variability in liver position, leaving outright CDH cases in which the liver is almost always herniated. The results obtained with the features computed in the manually segmented ROIs of the liver and lungs were compared with those obtained with those calculated in the nnU-Net segmented ROIs.

Since the MRI scans were very dissimilar in gray-level content, only shape features were used, discarding variables computed on the gray levels to avoid further image manipulation (intensity standardization). This choice left 22 features (Table 1), considering the liver and the lungs.

We trained and validated the models with a Leave One Patient Out (LOPO) scheme, in which each patient was chosen as the only element in the validation set, and the remaining patients built the training set. Classification quality was expressed as the area under the receiver operating characteristic (ROC) curve and by confusion matrices.

## Results

We enrolled 39 CDH cases, 30 with left and 9 with right side diaphragmatic defect. The dataset was quite balanced regarding liver herniation, with 22 *up* and 17 *down* cases. All the right-sided CDH cases were *up*.

**Fig. 1:**
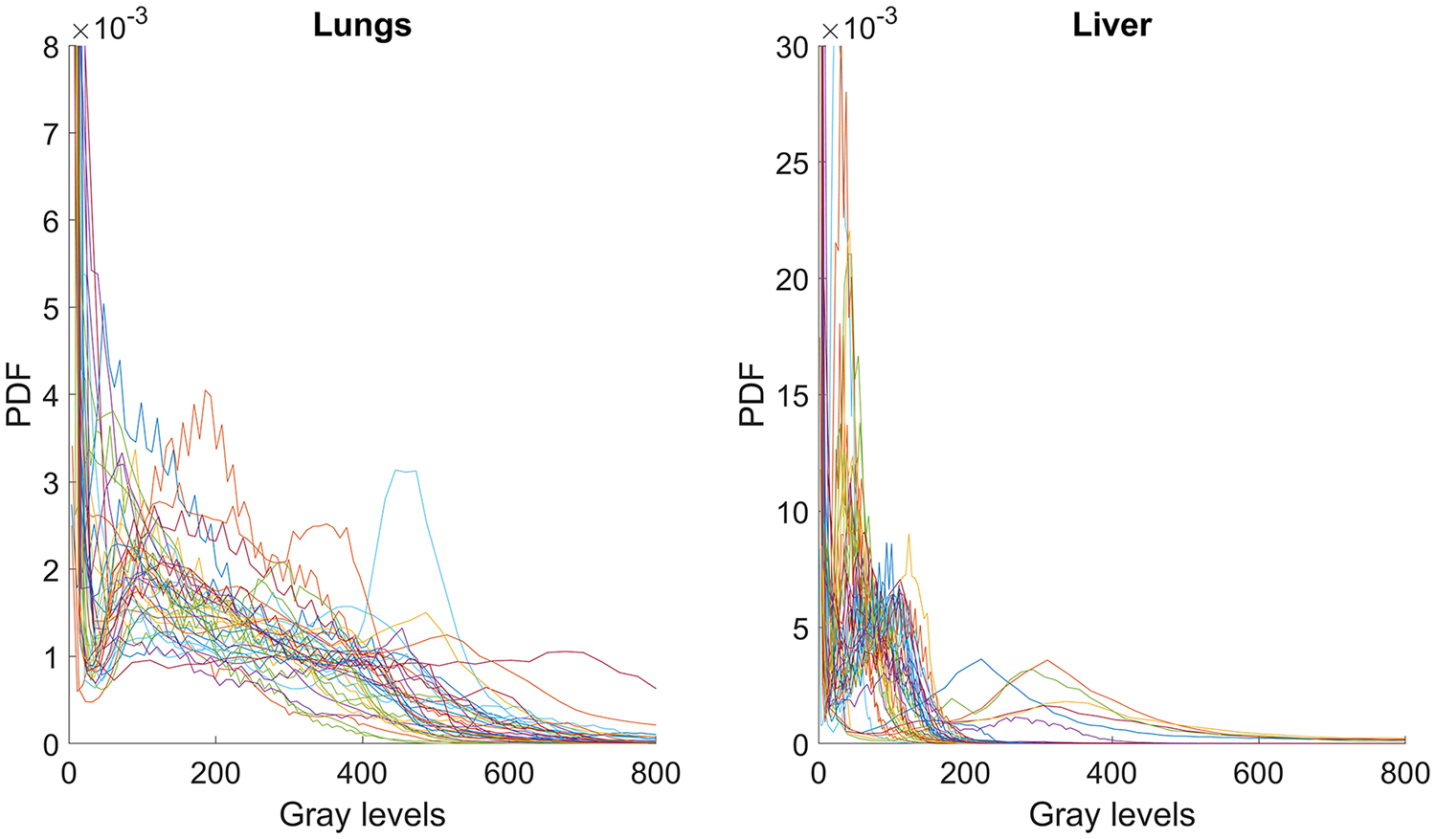
Inhomogeneity in the MR images. The gray-value histograms were calculated within the lung (left plots) and liver (right plots) manually segmented ROIs

The MR images were very inhomogeneous as to voxel size (the in-plane size was 0.21 mm to 0.78 mm, and the thickness was 3 mm or 6 mm) and gray level range (Fig. 1).

### Segmentation

**Fig. 2:**
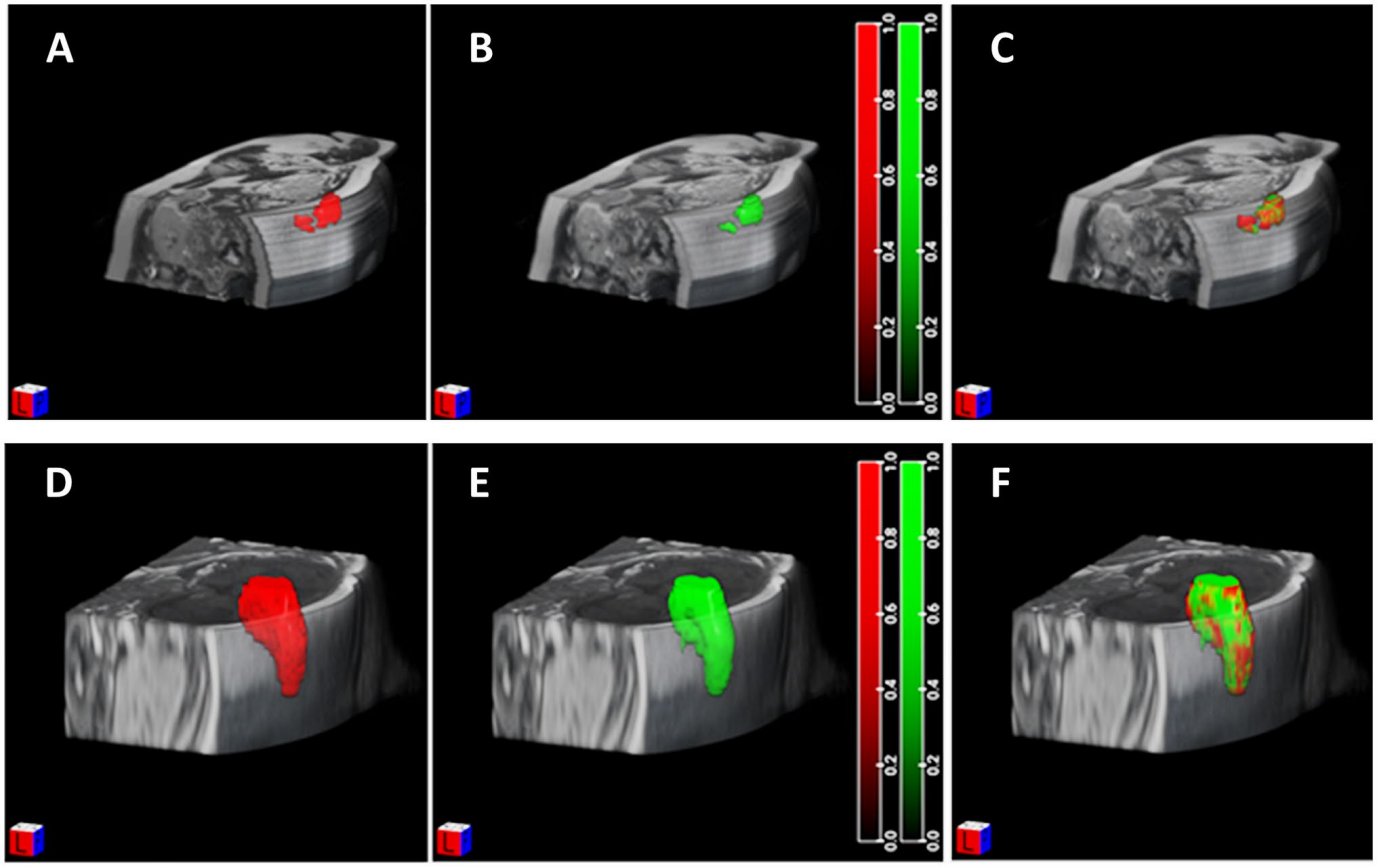
2D Segmentation results for the liver (top row) and lungs (bottom row). 3D manually segmented ROIs are shown in red **A**, **D**; automatic contouring is shown in green **B**, **E**. The overlaps of manual and automatic segmentations are shown in **C** and **F**

**Fig. 3:**
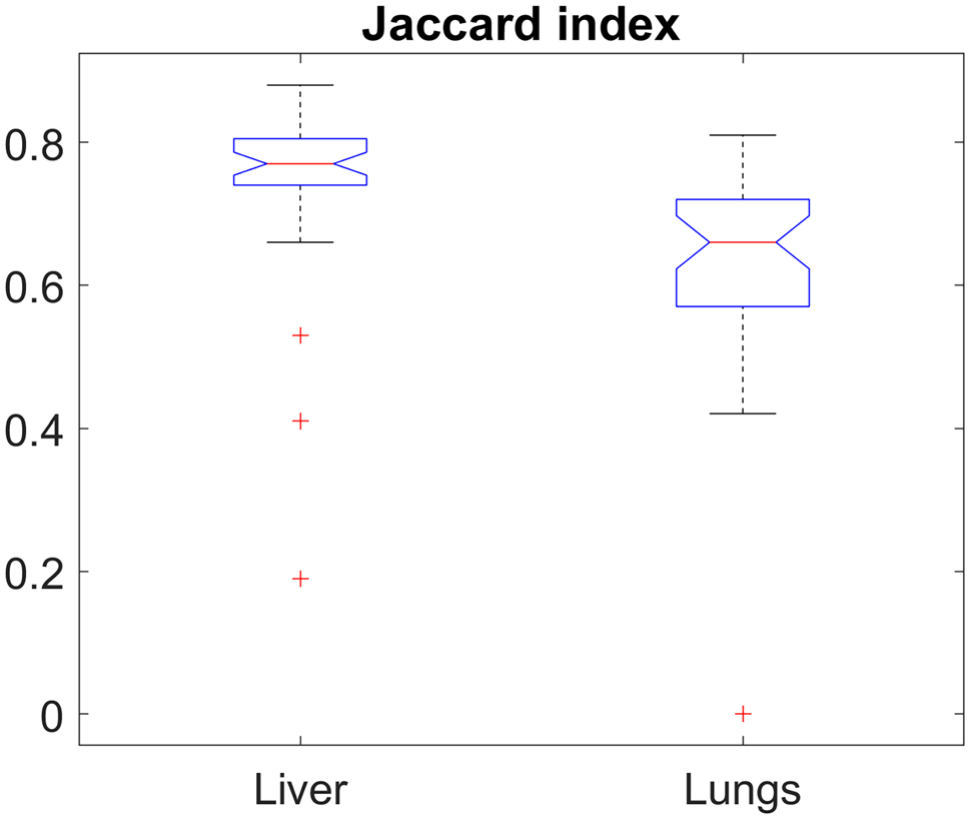
Boxplots report the mean Jaccard coefficient values for the lungs and liver

Segmentation results showed a very good accordance between manual and automatic methods. In Fig. 2, we reported an example of segmentation results of two single MRI cases of the liver and lungs, in which perfect accordance was observed. Nonetheless, quality varied for other images, and in some lung segmentation tests, one of the two lungs was lost during automatic segmentation. The Jaccard coefficient values for the whole dataset expressed as box plots are reported in Fig. 3. The average Jaccard coefficient for lung segmentation was 0.65, while liver segmentation showed better results with an average value of the Jaccard coefficient of 0.75. A Jaccard coefficient of 1 indicates perfect agreement, while a coefficient of 0 indicates no agreement.

### Reproducibility of pyradiomics features

Figures S1 and S2 (Supplemental Materials) show, respectively, for lungs and liver, the scatterplots of the features obtained for each variable, the values calculated in the manual ROIs (*x*-axis), and the corresponding values calculated in the automatic ROIs (*y*-axis). In case of perfect correspondence, the points should be located on the quadrant bisector.

To state the agreement between manual and automatic feature groups, we employed the Wilcoxon rank-sum test within each group of features. We also computed and examined ICCs across single features for testing interrater reliability. Table 2 provides results for single-measure ICCs under a two-way mixed model with absolute agreement.

**Table 2:** Intraclass correlation coefficients (ICCs) between radiomic features derived from manual and automatic segmentations for the liver (A) and the lungs (B). The Wilcoxon rank-sum test was executed for single features and across groups of features (e.g., shape and first order). Features with ICC < 0.40 were considered poorly reproducible and highlighted in light gray. A *p* value < 0.05 was considered statistically significant, and the corresponding rows were marked with one or more asterisks (*p < 0.05; **p < 0.01; ***p < 0.001)

			**Intraclass correlation**	**95% confidence interval**		***F* test**			
				**Lower bound**	**Upper bound**	**Value**	**df1**	**df2**	***p* value**
		**(A) Liver** ***All features p* = *0.5***							
**Shape** *p* < 0.001***	1	Mesh volume	0.930	0.870	0.962	27.244	38	39	< 0.001***
	2	Voxel volume	0.930	0.870	0.962	27.220	38	39	< 0.001***
	3	Surface area	0.850	0.553	0.937	18.172	38	8	< 0.001***
	4	Surface volume ratio	0.536	− 0.059	0.805	6.539	38	4	0.0432*
	5	Sphericity	0.347	− 0.089	0.704	6.113	38	2	0.1288
	6	Maximum 3D diameter	0.901	0.820	0.947	19.791	38	38	< 0.001***
	7	Major axis length	0.832	0.703	0.908	10.917	38	39	< 0.001***
	8	Minor axis length	0.917	0.848	0.955	23.358	38	39	< 0.001***
	9	Least axis length	0.734	0.546	0.851	6.384	38	38	< 0.001***
	10	Elongation	0.816	0.673	0.900	10.461	38	35	< 0.001***
	11	Flatness	0.619	0.381	0.780	4.204	38	39	< 0.001***
**First order** *p* < 0.05*	12	Energy	0.964	0.932	0.981	55.697	38	38	< 0.001***
	13	Total energy	0.964	0.932	0.981	55.697	38	38	< 0.001***
	14	Entropy	0.987	0.975	0.993	150.134	38	38	< 0.001***
	15	10th percentile	0.980	0.906	0.993	156.390	38	6	< 0.001***
	16	90th percentile	0.999	0.999	1.000	3.789.351	38	29	< 0.001***
	17	Interquartile range	0.886	0.682	0.951	22.904	38	10	< 0.001***
	18	Minimum	0.769	0.377	0.901	11.428	38	8	< 0.001***
	19	Maximum	0.614	0.348	0.783	4.752	38	26	< 0.001***
	20	Mean	0.996	0.987	0.998	733.124	38	10	< 0.001***
	21	Median	0.998	0.994	0.999	1.002.249	38	16	< 0.001***
	22	Mean absolute deviation	0.882	0.523	0.956	26.784	38	5	< 0.001***
	23	Robust mean absolute deviation	0.885	0.641	0.953	24.079	38	8	< 0.001***
	24	Root mean squared	0.997	0.993	0.999	837.940	38	16	< 0.001***
	25	Variance	0.816	0.375	0.928	16.237	38	6	0.0014**
	26	Skewness	0.539	0.272	0.729	3.301	38	38	< 0.001***
	27	Kurtosis	0.344	0.052	0.587	2.249	38	32	0.0107**
	28	Uniformity	0.985	0.972	0.992	132.381	38	38	< 0.001***
**GLCM** *p* < 0.01**	29	Autocorrelation	0.722	0.518	0.846	6.784	38	31	< 0.001***
	30	Cluster prominence	0.982	0.966	0.990	107.609	38	39	< 0.001***
	31	Cluster shade	0.975	0.952	0.987	75.764	38	38	< 0.001***
	32	Cluster tendency	0.988	0.977	0.994	160.895	38	38	< 0.001***
	33	Contrast	0.979	0.961	0.989	94.258	38	39	< 0.001***
	34	Correlation	0.646	0.421	0.796	4.705	38	39	< 0.001***
	35	Difference average	0.979	0.961	0.989	94.258	38	39	< 0.001***
	36	Difference entropy	0.988	0.978	0.994	167.361	38	38	< 0.001***
	37	Difference variance	0.984	0.969	0.991	121.408	38	39	< 0.001***
	38	Id	0.979	0.961	0.989	94.258	38	39	< 0.001***
	39	Idm	0.979	0.961	0.989	94.258	38	39	< 0.001***
	40	Idmn	0.979	0.961	0.989	94.258	38	39	< 0.001***
	41	Idn	0.979	0.961	0.989	94.258	38	39	< 0.001***
	42	Imc1	0.751	0.536	0.868	8.160	38	23	< 0.001***
	43	Imc2	0.980	0.959	0.990	108.675	38	29	< 0.001***
	44	Inverse variance	0.979	0.961	0.989	94.258	38	39	< 0.001***
	45	Joint average	0.723	0.519	0.847	6.789	38	31	< 0.001***
	46	Joint energy	0.987	0.976	0.993	152.688	38	38	< 0.001***
	47	Joint entropy	0.989	0.979	0.994	171.801	38	38	< 0.001***
	48	MCC	0.552	0.291	0.737	3.449	38	39	< 0.001***
	49	Maximum probability	0.986	0.973	0.992	135.579	38	38	< 0.001***
	50	Sum average	0.723	0.519	0.847	6.789	38	31	< 0.001***
	51	Sum entropy	0.989	0.979	0.994	178.548	38	38	< 0.001***
	52	Sum squares	0.988	0.976	0.993	156.073	38	38	< 0.001***
**GLRLM** ***p* = 0.42**	53	Gray level non-uniformity	0.917	0.779	0.963	30.586	38	12	< 0.001***
	54	Gray level non-uniformity normalized	0.986	0.974	0.993	151.640	38	36	< 0.001***
	55	Gray level variance	0.986	0.974	0.993	151.640	38	36	< 0.001***
	56	High gray level run emphasis	0.695	0.480	0.829	6.038	38	32	< 0.001***
	57	Long run emphasis	0.882	0.406	0.960	30.690	38	4	< 0.001***
	58	Long run high gray level emphasis	0.751	0.574	0.861	6.933	38	38	< 0.001***
	59	Long run low gray level emphasis	0.731	0.452	0.865	8.100	38	15	< 0.001***
	60	Low gray level run emphasis	0.695	0.480	0.829	6.038	38	32	< 0.001***
	61	Run entropy	0.672	0.186	0.857	7.958	38	7	0.0048**
	62	Run length non-uniformity	0.959	0.868	0.983	67.218	38	9	< 0.001***
	63	Run length non-uniformity normalized	0.764	0.156	0.914	14.238	38	4	0.0085**
	64	Run percentage	0.933	0.728	0.974	45.428	38	6	< 0.001***
	65	Run variance	0.937	0.884	0.967	30.222	38	38	< 0.001***
	66	Short run emphasis	0.732	− 0.034	0.914	17.045	38	3	0.0325*
	67	Short run high gray level emphasis	0.557	0.084	0.788	5.281	38	8	0.0109**
	68	Short run low gray level emphasis	0.809	0.667	0.895	9.644	38	39	< 0.001***
**GLSZM** ***p* = 0.17**	69	Gray level non-uniformity	0.918	0.849	0.956	22.752	38	38	< 0.001***
	70	Gray level non-uniformity normalized	0.412	0.116	0.641	2.393	38	39	0.0040**
	71	Gray level variance	0.412	0.116	0.641	2.393	38	39	0.0040**
	72	High gray level zone emphasis	0.505	0.226	0.706	2.993	38	38	< 0.001***
	73	Large area emphasis	0.809	0.604	0.905	11.539	38	18	< 0.001***
	74	Large area high gray level emphasis	0.532	0.270	0.722	3.381	38	38	< 0.001***
	75	Large area low gray level emphasis	0.779	0.599	0.881	8.993	38	28	< 0.001***
	76	Low gray level zone emphasis	0.505	0.226	0.706	2.993	38	38	< 0.001***
	77	Size zone non-uniformity	0.724	0.532	0.846	6.143	38	38	< 0.001***
	78	Size zone non-uniformity normalized	0.849	0.626	0.931	16.183	38	12	< 0.001***
	79	Small area emphasis	0.293	− 0.009	0.550	1.861	38	39	0.0285*
	80	Small area high gray level emphasis	0.434	0.150	0.654	2.611	38	38	0.0019**
	81	Small area low gray level emphasis	0.289	− 0.014	0.547	1.842	38	39	0.0306*
	82	Zone entropy	0.903	0.620	0.963	31.734	38	6	< 0.001***
	83	Zone percentage	0.902	0.816	0.948	20.833	38	33	< 0.001***
	84	Zone variance	0.552	0.287	0.738	3.400	38	38	< 0.001***
**GLDM** *p* < 0.01**	85	Dependence entropy	0.982	0.966	0.991	115.662	38	36	< 0.001***
	86	Dependence non-uniformity	0.918	0.843	0.957	25.392	38	32	< 0.001***
	87	Dependence non-uniformity normalized	0.957	0.851	0.983	66.081	38	8	< 0.001***
	88	Dependence variance	0.859	0.085	0.959	36.234	38	2	0.0151*
	89	Gray level non-uniformity	0.912	0.840	0.953	21.764	38	39	< 0.001***
	90	Gray level variance	0.985	0.972	0.992	132.381	38	38	< 0.001***
	91	High gray level emphasis	0.722	0.517	0.846	6.756	38	31	< 0.001***
	92	Large dependence emphasis	0.936	0.745	0.976	47.509	38	7	< 0.001***
	93	Large dependence high gray level emphasis	0.729	0.534	0.849	6.860	38	33	< 0.001***
	94	Large dependence low gray level emphasis	0.720	0.509	0.846	6.824	38	29	< 0.001***
	95	Low gray level emphasis	0.722	0.517	0.846	6.756	38	31	< 0.001***
	96	Small dependence emphasis	0.919	0.690	0.969	37.360	38	7	< 0.001***
	97	Small dependence high gray level emphasis	0.724	0.488	0.853	7.301	38	22	< 0.001***
	98	Small dependence low gray level emphasis	0.721	0.530	0.843	6.304	38	38	< 0.001***
**NGTDM** *p* < 0.01**	99	Busyness	0.870	0.767	0.930	14.404	38	39	< 0.001***
	100	Coarseness	0.742	0.545	0.859	7.424	38	30	< 0.001***
	101	Complexity	0.978	0.959	0.988	89.349	38	39	< 0.001***
	102	Contrast	0.962	0.929	0.980	52.607	38	39	< 0.001***
	103	Strength	− 0.071	− 0.386	0.253	0.871	38	38	0.6637
		**(B) Lungs** ***All features p* < *0.001***							
**Shape** *p* < 0.001***	1	Mesh volume	0.920	0.593	0.972	42.879	38	5	< 0.001***
	2	Voxel volume	0.919	0.583	0.972	42.935	38	5	< 0.001***
	3	Surface area	0.827	− 0.002	0.951	32.640	38	2	0.0253*
	4	Surface volume ratio	0.706	0.156	0.881	9.921	38	5	0.0074**
	5	Sphericity	0.283	− 0.101	0.620	3.796	38	3	0.1248
	6	Maximum 3D diameter	0.747	0.310	0.893	10.609	38	7	0.0016**
	7	Major axis length	0.786	0.431	0.907	12.125	38	8	< 0.001***
	8	Minor axis length	0.476	0.068	0.722	3.882	38	11	0.0114*
	9	Least axis length	0.648	0.243	0.831	6.552	38	10	0.0019**
	10	Elongation	0.464	0.184	0.676	2.930	38	34	< 0.001***
	11	Flatness	0.479	0.201	0.687	3.050	38	34	< 0.001***
**First order** ***p* = 0.07**	12	Energy	0.996	0.993	0.998	524.941	38	39	< 0.001***
	13	Total energy	0.996	0.993	0.998	524.941	38	39	< 0.001***
	14	Entropy	0.859	0.342	0.952	25.235	38	4	0.0029**
	15	10th percentile	0.901	0.221	0.971	49.339	38	3	0.0079**
	16	90th percentile	0.997	0.980	0.999	1.030.879	38	5	< 0.001***
	17	Interquartile range	0.829	0.157	0.945	23.584	38	3	0.0097**
	18	Minimum	0.632	0.055	0.847	7.803	38	5	0.0160*
	19	Maximum	0.892	0.804	0.942	17.740	38	39	< 0.001***
	20	Mean	0.979	0.700	0.994	233.179	38	3	< 0.001***
	21	Median	0.983	0.848	0.995	242.339	38	4	< 0.001***
	22	Mean absolute deviation	0.834	0.089	0.949	27.611	38	3	0.0146*
	23	Robust mean absolute deviation	0.831	0.142	0.946	24.527	38	3	0.0106*
	24	Root mean squared	0.989	0.956	0.996	266.500	38	8	< 0.001***
	25	Variance	0.818	0.108	0.942	23.030	38	3	0.0128*
	26	Skewness	0.517	0.244	0.714	3.409	38	33	< 0.001***
	27	Kurtosis	0.510	0.234	0.709	3.042	38	38	< 0.001***
	28	Uniformity	0.852	0.448	0.944	20.918	38	6	< 0.001***
**GLCM** *p* < 0.001***	29	Autocorrelation	0.961	0.829	0.986	80.237	38	6	< 0.001***
	30	Cluster prominence	0.767	0.374	0.900	11.275	38	8	< 0.001***
	31	Cluster shade	0.799	0.610	0.896	10.441	38	22	< 0.001***
	32	Cluster tendency	0.813	0.458	0.921	14.444	38	8	< 0.001***
	33	Contrast	0.932	0.793	0.971	39.938	38	10	< 0.001***
	34	Correlation	0.448	− 0.055	0.734	4.483	38	5	0.0441*
	35	Difference average	0.932	0.793	0.971	39.938	38	10	< 0.001***
	36	Difference entropy	0.923	0.633	0.973	43.062	38	5	< 0.001***
	37	Difference variance	0.929	0.741	0.972	40.968	38	7	< 0.001***
	38	Id	0.932	0.793	0.971	39.938	38	10	< 0.001***
	39	Idm	0.932	0.793	0.971	39.938	38	10	< 0.001***
	40	Idmn	0.932	0.793	0.971	39.938	38	10	< 0.001***
	41	Idn	0.932	0.793	0.971	39.938	38	10	< 0.001***
	42	Imc1	0.237	− 0.068	0.512	2.137	38	13	0.0708
	43	Imc2	0.706	0.047	0.892	11.674	38	4	0.0177*
	44	Inverse variance	0.932	0.793	0.971	39.938	38	10	< 0.001***
	45	Joint average	0.961	0.841	0.985	78.679	38	7	< 0.001***
	46	Joint energy	0.878	0.556	0.953	24.568	38	6	< 0.001***
	47	Joint entropy	0.885	0.494	0.959	28.722	38	5	< 0.001***
	48	MCC	0.398	− 0.051	0.686	3.654	38	7	0.0441*
	49	Maximum probability	0.873	0.575	0.949	22.679	38	7	< 0.001***
	50	Sum average	0.961	0.841	0.985	78.679	38	7	< 0.001***
	51	Sum entropy	0.876	0.457	0.955	26.662	38	5	0.0011**
	52	Sum squares	0.850	0.514	0.939	18.960	38	7	< 0.001***
**GLRLM** ***p* = 0.37**	53	Gray level non-uniformity	0.855	0.180	0.955	29.323	38	3	0.0088**
	54	Gray level non-uniformity normalized	0.863	0.425	0.950	23.981	38	5	0.0013**
	55	Gray level variance	0.863	0.425	0.950	23.981	38	5	0.0013**
	56	High gray level run emphasis	0.926	0.430	0.978	58.323	38	3	0.0026**
	57	Long run emphasis	0.868	0.256	0.958	30.871	38	3	0.0058**
	58	Long run high gray level emphasis	0.887	0.262	0.966	38.608	38	3	0.0060**
	59	Long run low gray level emphasis	0.889	0.609	0.956	26.473	38	7	< 0.001***
	60	Low gray level run emphasis	0.926	0.430	0.978	58.323	38	3	0.0026**
	61	Run entropy	0.865	0.756	0.927	13.452	38	38	< 0.001***
	62	Run length non-uniformity	0.761	0.021	0.922	17.611	38	3	0.0216*
	63	Run length non-uniformity normalized	0.834	0.421	0.936	18.093	38	6	< 0.001***
	64	Run percentage	0.894	0.204	0.969	45.455	38	3	0.0084**
	65	Run variance	0.925	0.792	0.967	34.864	38	11	< 0.001***
	66	Short run emphasis	0.832	0.423	0.934	17.681	38	6	< 0.001***
	67	Short run high gray level emphasis	0.545	0.125	0.770	4.742	38	10	0.0062**
	68	Short run low gray level emphasis	0.887	0.619	0.955	25.350	38	7	< 0.001***
**GLSZM** ***p* = 0.38**	69	Gray level non-uniformity	0.780	0.620	0.878	8.245	38	39	< 0.001***
	70	Gray level non-uniformity normalized	0.613	0.377	0.776	4.275	38	39	< 0.001***
	71	Gray level variance	0.613	0.377	0.776	4.275	38	39	< 0.001***
	72	High gray level zone emphasis	0.860	0.750	0.924	13.350	38	39	< 0.001***
	73	Large area emphasis	0.512	0.243	0.709	3.296	38	35	< 0.001***
	74	Large area high gray level emphasis	0.526	0.258	0.719	3.445	38	35	< 0.001***
	75	Large area low gray level emphasis	0.611	0.372	0.774	4.328	38	37	< 0.001***
	76	Low gray level zone emphasis	0.860	0.750	0.924	13.350	38	39	< 0.001***
	77	Size zone non-uniformity	0.672	0.459	0.813	5.174	38	39	< 0.001***
	78	Size zone non-uniformity normalized	0.347	0.041	0.595	2.396	38	24	0.0132*
	79	Small area emphasis	0.500	0.229	0.700	3.149	38	37	< 0.001***
	80	Small area high gray level emphasis	0.850	0.731	0.918	12.013	38	38	< 0.001***
	81	Small area low gray level emphasis	0.642	0.398	0.798	5.081	38	30	< 0.001***
	82	Zone entropy	0.464	− 0.004	0.731	4.198	38	7	0.0260*
	83	Zone percentage	0.628	0.392	0.787	4.302	38	38	< 0.001***
	84	Zone variance	0.600	0.355	0.768	4.255	38	35	< 0.001***
**GLDM** ***p* = 0.15**	85	Dependence entropy	0.911	0.372	0.973	47.992	38	3	0.0034**
	86	Dependence non-uniformity	0.980	0.962	0.989	96.663	38	38	< 0.001***
	87	Dependence non-uniformity normalized	0.912	0.413	0.972	46.063	38	3	0.0026**
	88	Dependence variance	0.797	0.500	0.908	12.105	38	10	< 0.001***
	89	Gray level non-uniformity	0.962	0.903	0.983	66.818	38	14	< 0.001***
	90	Gray level variance	0.852	0.448	0.944	20.918	38	6	< 0.001***
	91	High gray level emphasis	0.958	0.791	0.985	77.854	38	5	< 0.001***
	92	Large dependence emphasis	0.895	0.161	0.970	49.335	38	2	0.0105*
	93	Large dependence high gray level emphasis	0.948	0.364	0.986	104.920	38	2	0.0047**
	94	Large dependence low gray level emphasis	0.965	0.934	0.981	57.561	38	38	< 0.001***
	95	Low gray level emphasis	0.958	0.791	0.985	77.854	38	5	< 0.001***
	96	Small dependence emphasis	0.847	0.729	0.917	11.952	38	39	< 0.001***
	97	Small dependence high gray level emphasis	0.736	0.552	0.852	6.753	38	38	< 0.001***
	98	Small dependence low gray level emphasis	0.874	0.773	0.932	14.544	38	38	< 0.001***
**NGTDM** *p* = 0.001***	99	Busyness	0.818	0.642	0.906	11.637	38	22	< 0.001***
	100	Coarseness	0.050	− 0.248	0.348	1.111	38	38	0.3729
	101	Complexity	0.927	0.783	0.969	36.326	38	10	< 0.001***
	102	Contrast	0.907	0.778	0.957	25.936	38	15	< 0.001***
	103	Strength	0.067	− 0.230	0.361	1.151	38	39	0.3321

Based on the approach chosen by Owens et al., we then classified the 103 features into four groups according to their ICC values having excellent (ICC ≥ 0.75), good (0.60 ≤ ICC < 0.75), fair (0.40 ≤ ICC < 0.60), or poor reproducibility (ICC < 0.40) [29]. Results are visually reported in Fig. S3 (Supplemental Materials) with a heat map. Of the 103 features, 46 (45%) showed excellent reproducibility, 11 (11%) exhibited good reproducibility, 22 (21%) showed fair reproducibility, and in 24 features (23%), reproducibility was poor.

### Machine learning

As previously stated, only MRI shape features were used to automatically classify up vs. down liver herniation. In order to test whether the features considered at high reproducibility were more predictive in detecting liver herniation than the others, we also used ICC values as cut-offs for feature selection. In the first test, all the features were used without exclusion (case 1: no selection). In subsequent attempts, three thresholds were selected: 0.60, 0.70, and 0.75 (cases 2 to 4), and only the radiomic features with ICC values not lower than the threshold were considered. The features of the lung and liver were included for each specific case, and the corresponding results are shown in Table 3.

**Table 3:** Various classification tests without and with feature selection. Case 1 included all the shape features, while cases 2 to 4 selected features based on three different cut-offs on the ICCs values (see “Machine learning”). The last two columns report AUCs obtained for manual and automatic ROIs

		Liver										Lungs											
Case	Threshold	Mesh volume	Surface area	Surface volume ratio	Sphericity	Maximum 3D diameter	Major axis length	Minor axis length	Least axis length	Elongation	Flatness	Mesh volume	Surface area	Surface volume ratio	Sphericity	Maximum 3D diameter	Major axis length	Minor axis length	Least axis length	Elongation	Flatness	AUC for manual ROIs	AUC for automatic ROIs
1	None	X	X	X	X	X	X	X	X	X	X	X	X	X	X	X	X	X	X	X	X	0.86	0.84
2	0.60	X	X			X	X	X	X	X	X	X	X	X		X	X		X			0.77	0.80
3	0.70	X	X			X	X	X	X	X	X	X	X			X	X					0.76	0.75
4	0.75	X	X			X	X	X		X	X	X	X				X					0.75	0.74

**Fig. 4:**
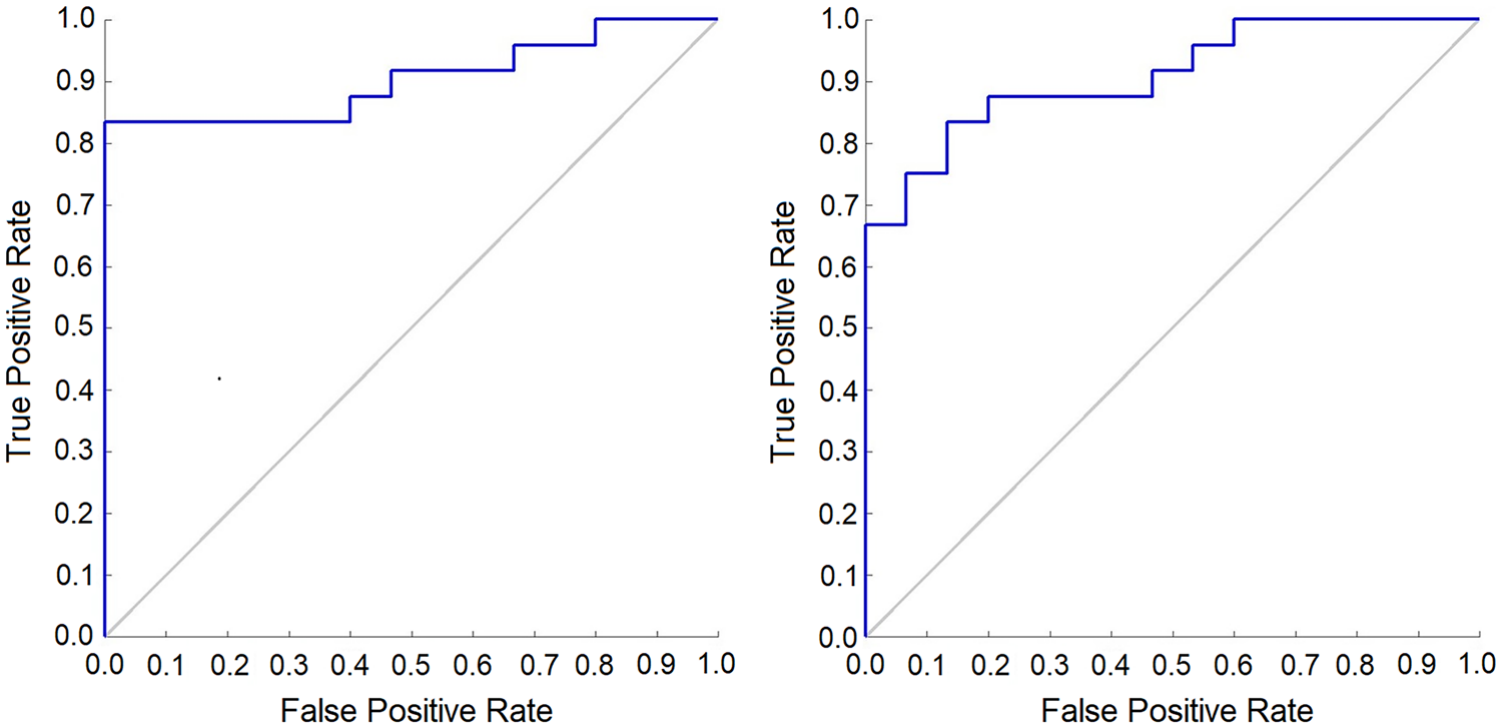
ROC curves for liver herniation prediction with SVM classifier and shape features. Left: manually segmented ROIs, right: nnU-Net segmentations

The best results were obtained without feature selection. Figure 4 shows the ROC curves for liver herniation (up/down) acquired by the best-tested classifier (a linear SVM). Without feature selection, the AUC obtained for the dataset of manually segmented ROIs and the one for the automatically segmented ROIs were equal to 0.86 and 0.84, respectively. The confusion matrices (cm) obtained and the corresponding values for sensitivity, specificity, and accuracy are reported in Table 4.

**Table 4:** Metrics of performance obtained for manually vs. automatically segmented ROIs

	cm (TP, FP, FN, TN)	Accuracy	Sensitivity	Specificity
Manual ROIs	$\left(15, 2, \mathrm{3,10}\right)$	83%	83%	83%
Automatic ROIs	$\left(\mathrm{16,1}, 5, 8\right)$	80%	76%	89%

*cm* confusion matrices, *FN* false negatives, *FP* false positives, *TN* true negatives, *TP* true positives

## Discussion

In newborns with CDH, automatic segmentation of the fetal lung and liver is feasible and shows high accordance with manual results. To the best of our knowledge, this represents the first attempt to apply an automatic segmentation system for fetuses with CDH, aiming to standardize the assessment of lung and liver volume and provide a reliable automatic prediction of liver herniation, which represent two main prognostic factors for postnatal outcome.

The segmentation software selected for this work was nnU-Net, a general-purpose 3D biomedical image segmentation tool. nnU-Net is designed to automatically deal with the dataset diversity found in the medical domain due to imaging modality, image sizes, voxel spacing (isotropic/anisotropic), pixel intensity (quantitative and standard as in computed tomography or essentially qualitative and non-standard as in MRI). This method demonstrates the flexibility most segmentation frameworks, designed on specific image types and properties, do not allow. Moreover, nnU-Net automates the key decisions for designing a segmentation system for a given dataset, significantly speeding up application development. Furthermore, if any improvement in segmentation quality is desired, the nnU-Net modular structure allows easy integration of new architectures and methods. nnU-Net relies on Python v3 and PyTorch and needs NVIDIA Compute Unified Device Architecture (CUDA) for most operations [24]. The quality of segmentation obtained with nnU-Net in the dataset of interest for this work was quite good, as demonstrated by the values of the Jaccard coefficients. An average Jaccard coefficient of 0.65 for lung segmentation suggested that, on average, the overlap between the algorithm segmentation and the ground truth segmentation was 65%. This meant good accuracy, as more than half of the segmented area was correctly overlapped with the actual area. The higher average Jaccard coefficient of 0.75 for liver segmentation indicated even better accuracy, with 75% overlap. Segmentation of the liver was better than the lung one: such a result was expected because of the larger organ volume compared to the lungs, which are even smaller in these patients due to the mechanism of the disease. Some values of the Jaccard coefficient were very large, but this was not true for all the patients.

After directly comparing the ROIs produced manually with those segmented with nnU-Net, we decided to compare the pyradiomics features computed in the automatically segmented ROIs with those extracted from the manual ROIs, as an indirect and practical test of segmentation quality. The rationale behind this test was that manual segmentation is a very time-consuming process that can hardly be applied to large datasets, so there is an interest in ascertaining if features extracted from ROIs obtained by automatic segmentation could produce results as accurate and useful as those extracted from regions drawn by manual segmentation.

For this purpose, we applied correlation and reproducibility tests to the two sets of features. Various techniques were employed to test for feature reproducibility between manual and automatic ROIs. Figures S1, S2, and S3 qualitatively show that some variables are reproducible so that their use can be granted, while others are not. This is particularly true for tiny lungs, and the Wilcoxon rank-sum test was used for the significance check. Our tests demonstrated that the two groups were significantly correlated and showed good agreement as measured by ICC.

A further indirect test of segmentation quality was performed by building a ML application for binary liver-herniation prediction/classification based on the features computed from manually or automatically segmented ROIs of the lungs and the livers. It was remarked that the MR images were very different in grayscale, so using features based on gray values would have needed some procedure of intensity standardization. For this reason, we avoided further image manipulation and only used shape features, discarding variables computed on the gray levels. Various classifiers were employed with similar results, and the highest performing was a linear SVM, which was trained on both feature sets (shape features extracted from manual vs. automatic ROIs). The two sets yielded similar (quite large) discrimination power between the up and down liver, as measured by the AUC value. Also, the shapes of the ROC curves were quite similar. This result suggested that the automatic segmentations produced by nnU-Net can be practically employed in ML applications. Even using less reproducible features helped classify liver up/down conditions, as it was found that selecting only highly reproducible features decreased classification quality. It is also interesting that when the whole sets of features were used (from manual vs. automatic ROIs), there was almost no difference in AUC between the two sets (AUC = 0.86 and 0.84, respectively), while feature selection led to a disparity in AUC with larger values for the set of features extracted from the automatic ROIs. Shape features, being based only on the ROI contour, might potentially be more deeply affected by segmentation errors, so the fact that AUC did not decrease from manual to automatic ROIs, and even increased when a partial dataset was chosen, is particularly significant and proves the goodness of the automatic segmentation.

Finally, it is noteworthy that, though the described ML application started as a convenient means for assessing feature reproducibility and thus as a test of goodness for nnU-Net segmentation, it was also a helpful result per se, suggesting that such reliable classification is feasible.

However, the limited number of cases has to be considered when considering these results, as CDH is a rare disease, which leads to a limited dataset of images available.

To increase the study population, collaboration with other institutions and the inclusion of future cases could be considered. Moreover, data augmentation by the generation of synthetic data can be a way to artificially increase dataset cardinality during training, which may have a positive impact on the segmentation of lungs and livers from the original data.

Traditional forms exist (e.g., the application of spatial transformations to the images), and more recent approaches based on neural networks look promising, particularly for tiny datasets [30].

Another critical aspect, mainly concerning the ML results, depends on data inhomogeneity, specifically the lack of a standard grayscale in the images. To overcome this limit, we chose to discard ML features based on the gray-level content of ROIs. Image standardization (i.e., wisely transforming the images to a common gray-level scale) is possible, though it is very delicate and demanding. The advantage would be that after standardization, gray-level-based features—at least those with good reproducibility from manual to automatic ROIs—could also be used for classification purposes to increase ML quality. It is also possible that image standardization may lead to an increase in nnU-Net segmentation quality, helping the segmentation algorithms.

Despite these limitations, the findings of our research are encouraging. The definition of an automatic segmentation software tool specifically designed for the fetal lung and liver would be relevant to clinical practice. Since CDH assessment is largely based on prenatal imaging, automatic segmentation would be key in simplifying and standardizing the diagnostic process. Moreover, it would provide more accurate imaging data for developing robust algorithms and tools for the early prediction of postnatal outcomes.

Artificial intelligence-based prediction systems are proving to greatly support the interpretation of clinical data and images of various conditions in the NICUs. For example, AI systems have been successfully developed to analyze retinal images for diagnosing retinopathy of prematurity and plus disease, where some subtle and fine signals may escape the human eye [31–34]. AI models could identify complex patterns and associations in the volume of data available in preterm infant EHRs that traditional statistical methods or human experts may miss. These models can facilitate early detection of complications such as sepsis and necrotizing enterocolitis [35–38].

AI enables data integration from multiple sources, such as imaging modalities and clinical features. As a future perspective, fetal MRI and US data should be integrated with fetal-maternal clinical variables automatically extracted from electronic medical records. Identifying critical factors and assessing the relationship between clinical-radiologic variables and patient outcomes might help to further elucidate the major determinants of CDH pathophysiology, especially postnatal pulmonary hypertension. Through an integrated multimodal analysis, the early detection of key features could enable the building of forecasting prognostic algorithms and provide a unique advancement in managing fetuses and neonates with CDH, ultimately improving the overall quality of care. For example, parental counseling would be more accurate, helping parents to understand the pathological condition better and feel more involved in the care process. Prenatal risk stratification is also crucial for the appropriate selection of FETO candidates. After birth, algorithms may be able to anticipate critical events and guide timely interventions, such as determining the optimal timing for surgery or indicating the onset of complications. Patients at high risk of ECMO could also be identified. In addition, more rational resource allocation and cost-effective management could be facilitated.

## Conclusions

Within the limitations of this study, automatic MRI segmentation of the lungs and liver of CDH fetuses through nnU-Net is feasible, with good reproducibility of pyradiomics features. In addition, a machine learning approach for predicting liver herniation offers good reliability.

Our results could open the way to new applications of artificial intelligence in the neonatal field to standardize prenatal assessment and provide a reliable automatic tool for prognostic evaluation in CDH patients.

### Supplementary Information

Below is the link to the electronic supplementary material.

Supplementary file1 (DOCX 2041 KB)

## Data Availability

The data employed in this article may be available for sharing upon request. Interested parties may contact the authors for access.
