# Supplementary material for: Congenital diaphragmatic hernia: automatic lung and liver MRI segmentation with nnU-Net, reproducibility of pyradiomics features, and a machine learning application for the classification of liver herniation
Source: Eur J Pediatr. 2024 Feb 28;183(5):2285–300. doi: 10.1007/s00431-024-05476-9 (PMC11035462; doi:10.1007/s00431-024-05476-9)
Supplement: Supplementary file 1 — Supplementary file1 (DOCX 2041 KB) [file 431_2024_5476_MOESM1_ESM.docx]

**Supplemental materials**


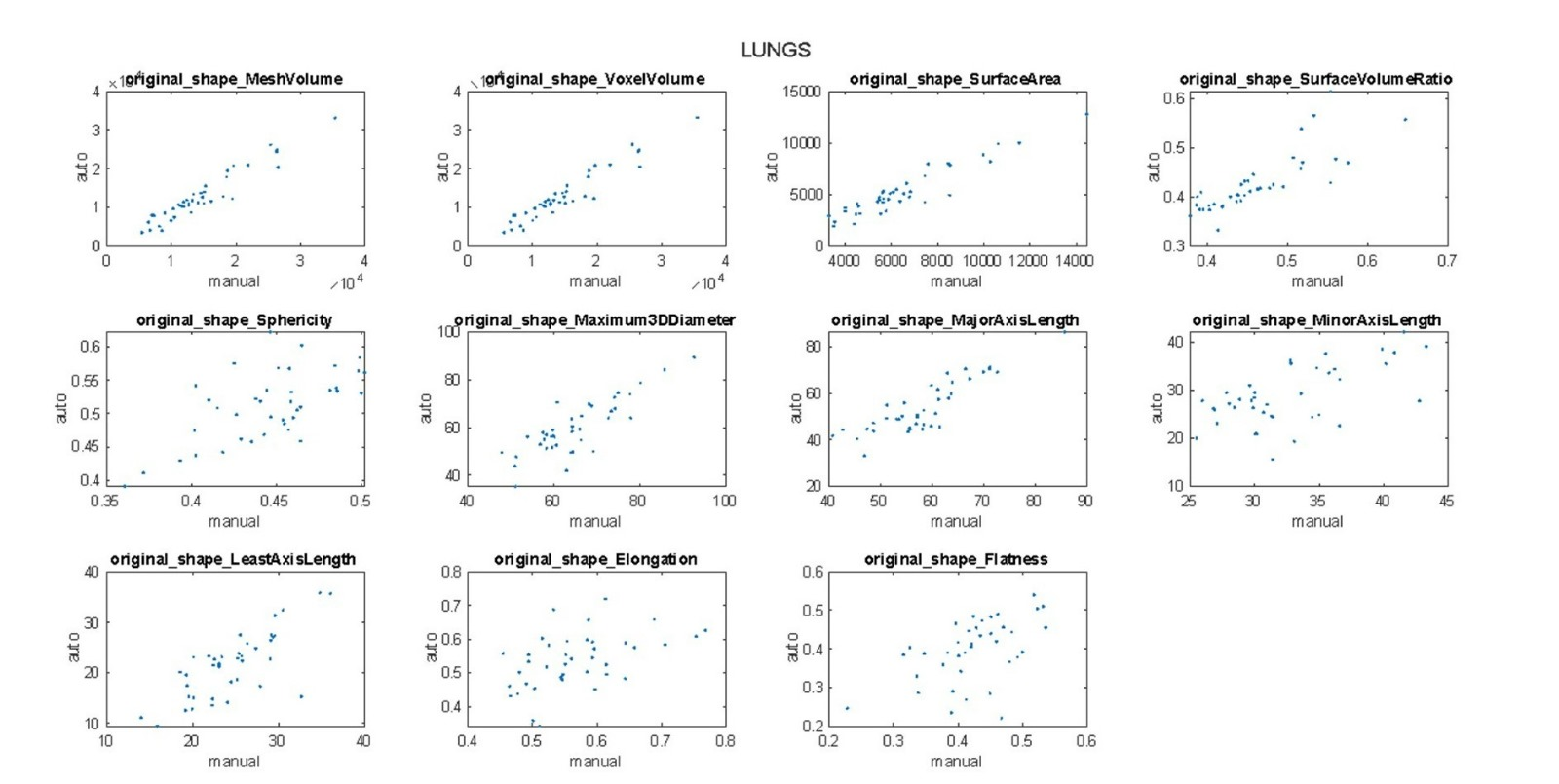


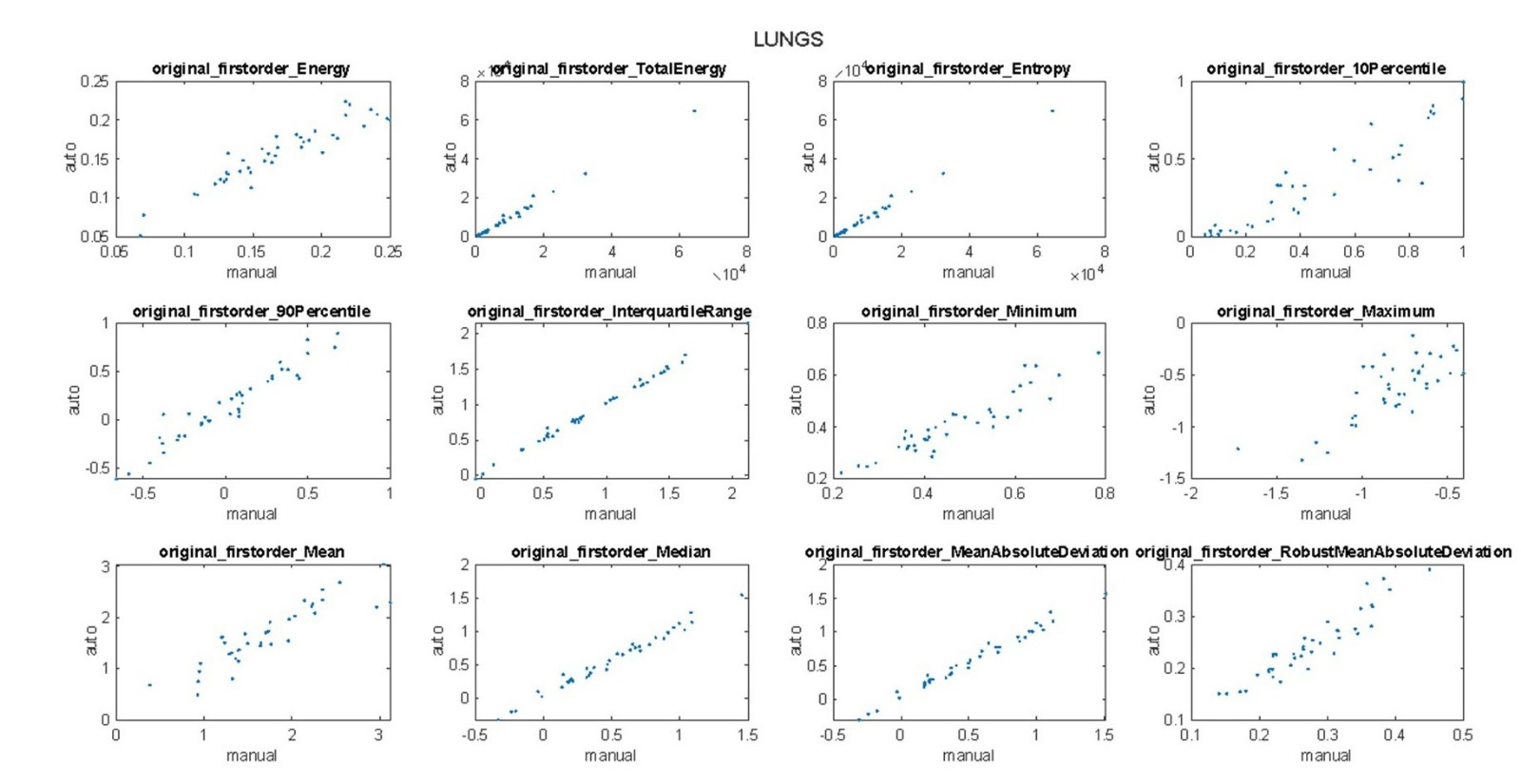


**Figure S1.** Scatter plots of feature values computed in automatic (y-axis) and manual (x-axis) ROIs for Lungs. Reproducibility is good when the points lie on the quadrant bisector or near it.


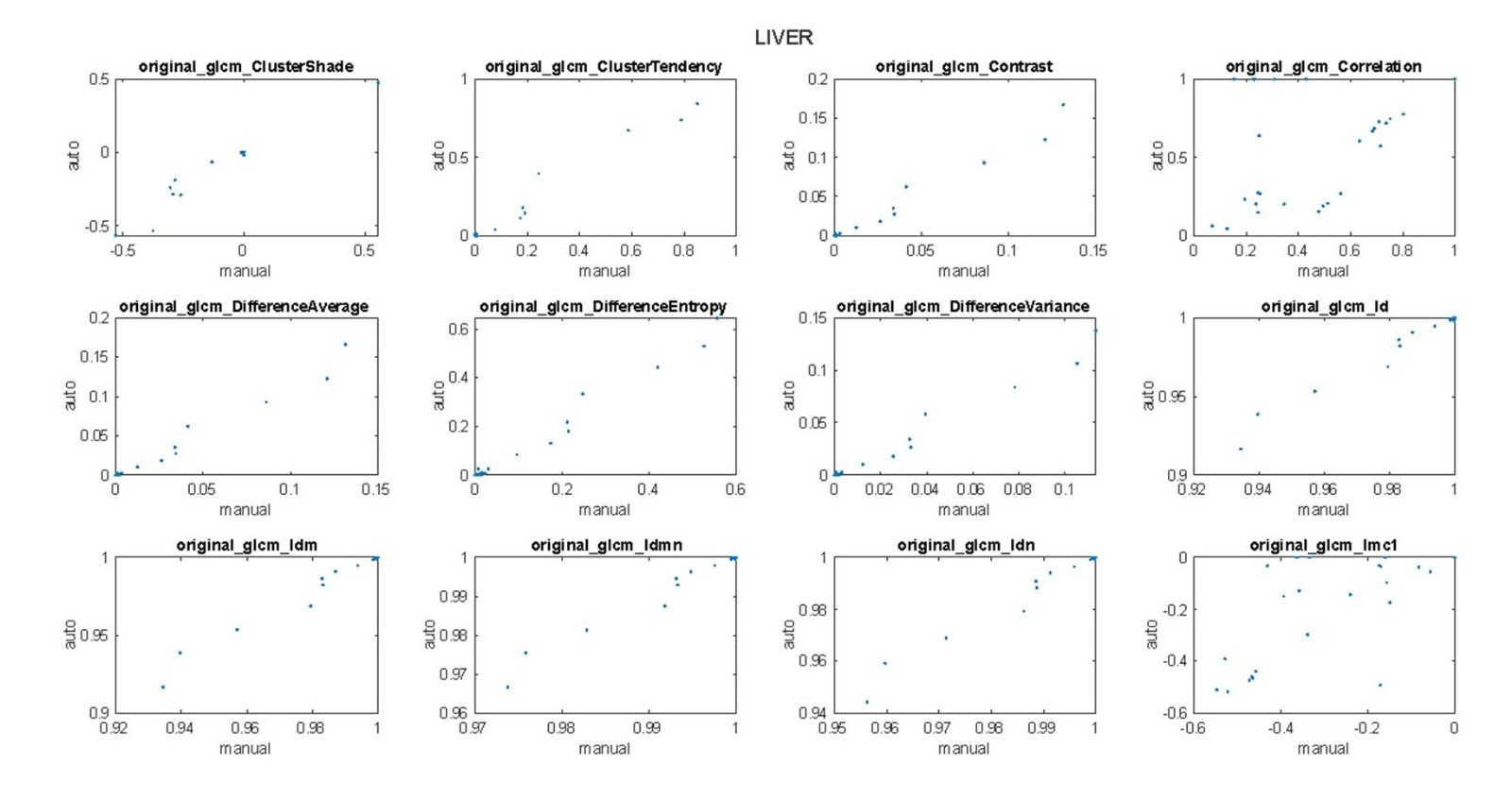


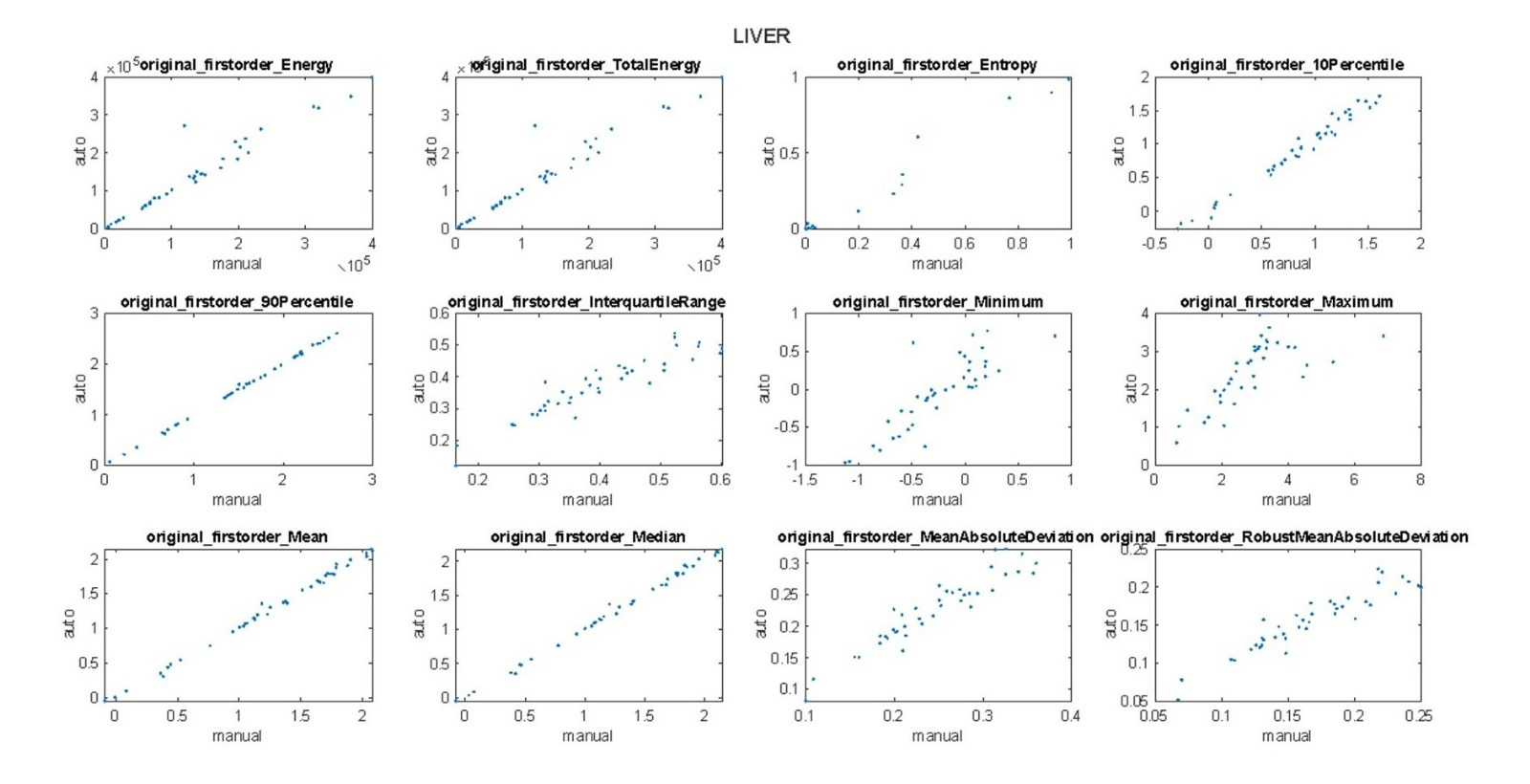


**Figure S2.** Scatter plots of feature values computed in automatic (y-axis) and manual (x-axis) ROIs for Liver. Reproducibility is good when the points lie on the quadrant bisector or near it.

*
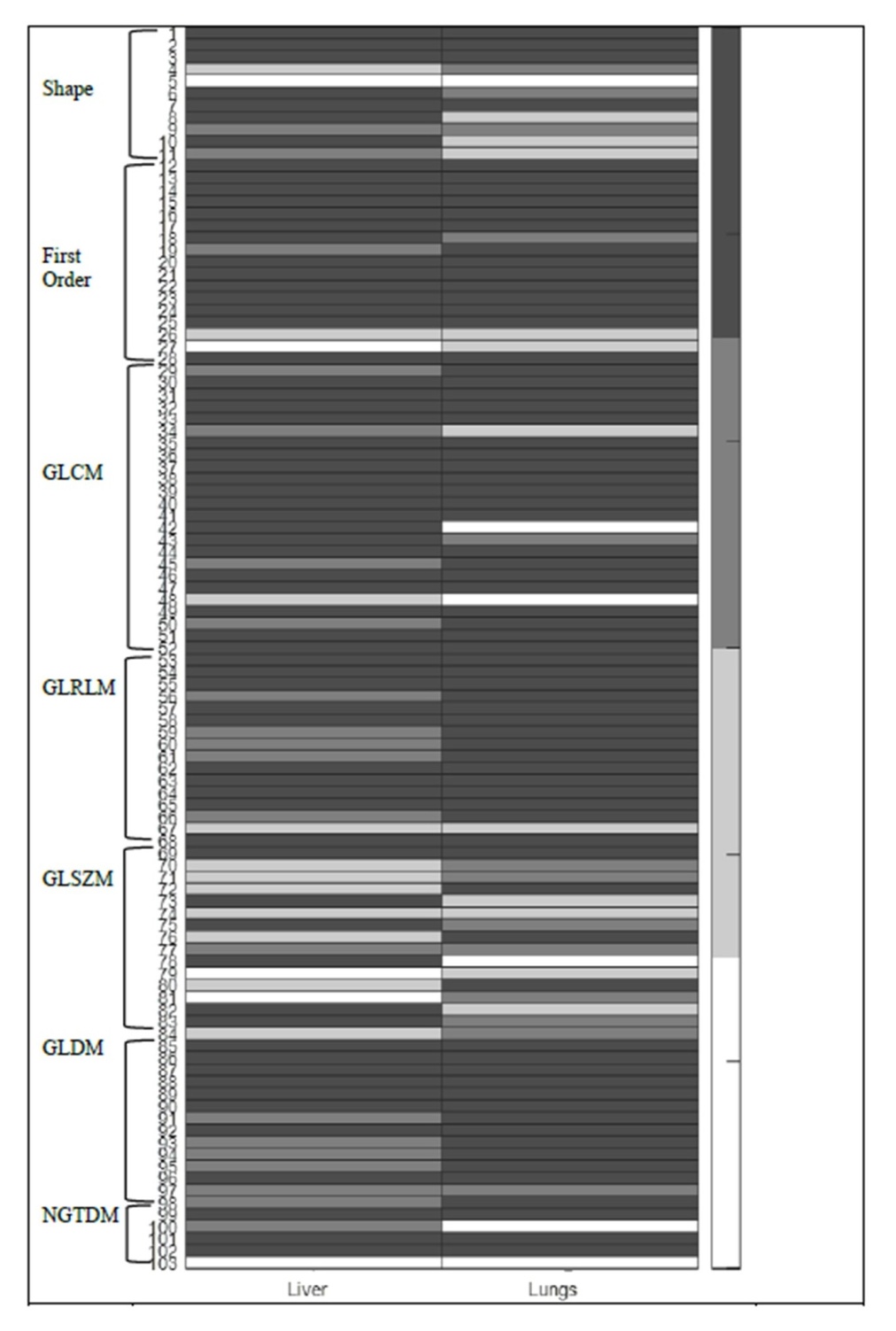
*

**Figure S3.** Heatmap showing ICC classification of each radiomic feature class for liver (left side) and lungs (right side). Dark gray, medium gray, light gray, and white denote the ICC classification of excellent (ICC ≥ 0.75), good (0.60 ≤ ICC < 0.75), fair (0.4 ≤ ICC < 0.60), and poor (ICC < 0.4) reproducibility, respectively. The feature name corresponding to each index value can be deduced from Table 2.
